# Histrelin Implants for Suppression of Puberty in Youth with Gender Dysphoria: A Comparison of 50 mcg/Day (Vantas) and 65 mcg/Day (SupprelinLA)

**DOI:** 10.1089/trgh.2020.0055

**Published:** 2021-02-15

**Authors:** Johanna Olson-Kennedy, Laer H. Streeter, Robert Garofalo, Yee-Ming Chan, Stephen M. Rosenthal

**Affiliations:** ^1^ The Center for Transyouth Health and Development, Children's Hospital Los Angeles, Los Angeles, California, USA.; ^2^ Department of Pediatrics, University of Southern California Keck School of Medicine, Los Angeles, California, USA.; ^3^ University of Southern California Keck School of Medicine, Los Angeles, California, USA.; ^4^ Center for Gender, Sexuality and HIV Prevention, Lurie Children's Hospital, Northwestern Feinberg School of Medicine, Chicago, Illinois, USA.; ^5^ The Potocsnak Family Division of Adolescent and Young Adult Medicine, Ann & Robert H. Lurie Children's Hospital of Chicago, Northwestern University Feinberg School of Medicine, Chicago, Illinois, USA.; ^6^ Gender Management Services, Boston Children's Hospital, Harvard Medical School, Boston, Massachusetts, USA.; ^7^ Child and Adolescent Gender Center, Benioff Children's Hospital-San Francisco, University of California San Francisco, San Francisco, California, USA.

**Keywords:** gender dysphoria, puberty suppression, puberty blockers, transgender youth

## Abstract

**Purpose:** Development of incongruent secondary sex characteristics in transgender youth can intensify or trigger the onset of gender dysphoria. Guidelines from professional organizations recommend gonadotropin-releasing hormone agonists, including histrelin implants (Vantas and SupprelinLA) to suppress endogenous puberty. Although Vantas does not have a pediatric indication, it is anecdotally being used in pediatric gender centers throughout the United States because of its substantially lower cost. This retrospective study aimed to determine if both implants were effective in suppressing the hypothalamic–pituitary–gonadal axis in early-to-mid pubertal youth with gender dysphoria.

**Methods:** Youth with gender dysphoria receiving care at the Center for Transyouth Health and Development at Children's Hospital Los Angeles (CHLA) or participants from an ongoing observational trial with a histrelin implant placed for pubertal suppression at Tanner stage 2 or 3 were included. Sex steroid (testosterone or estradiol) and gonadotropin measurements at baseline (T0) and then 2 to 12 months following implant placement (T1) were abstracted from medical records.

**Results:** Of the 66 eligible participants, 52% were designated female at birth. Most participants were white (60.6%). Twenty participants (30.3%) had a Vantas implant and 46 (69.7%) had a SupprelinLA implant. Mean age of insertion was 11.3 years. Gonadotropin and sex steroid levels were significantly decreased at T1 (2–12 months after insertion of implant), with no differences between implants.

**Conclusion:** These results indicate that both implants are effective in suppressing puberty in early-to-mid pubertal youth with gender dysphoria. These data may inform decisions about insurance coverage of Supprelin and/or Vantas for youth with gender dysphoria.

## Background

Development of secondary sex characteristics that are incongruent with gender identity can intensify gender dysphoria or trigger the initial onset of gender dysphoria in transgender youth starting puberty.^1,2^ Transgender youth experiencing gender dysphoria are at increased risk for anxiety, depression, suicide, and substance use compared with their peers.^3^ Undesired secondary sex characteristics might include a laryngeal prominence, deepening of the voice, and tall stature in transfeminine youth (those designated male at birth); and breast development, menstruation, and short stature in transmasculine individuals (those designated female at birth).

In 2006, a team of experts from the Netherlands outlined the use of gonadotropin-releasing hormone agonists (GnRHa's) for the suppression or delay of undesired endogenous puberty in youth with gender dysphoria.^1^ In addition to eliminating the stress of developing irreversible secondary sexual characteristics of a body with which they may not identify, endogenous puberty suppression may also eliminate the need for certain gender-affirming surgeries later in life, such as chest reconstruction and chondrolaryngoplasty (commonly called tracheal shave). Additionally, the use of pubertal suppression to pause sexual development allows additional time for parents and caregivers to manage their own process of understanding their child's gender identity so that they can more appropriately support their child.^1,2^

To avoid the development of undesired endogenous secondary sex characteristics in youth with gender dysphoria, the Endocrine Society and cosponsoring organizations recommend that GnRHa's be administered to transgender youth at the earliest stages of puberty (Tanner 2–3).^3,4^ GnRHa's are also provided to older youth (Tanner stages 4–5) in combination with exogenous sex hormones for the purposes of inducing amenorrhea in transmasculine individuals and suppressing testosterone secretion to augment the effects of estrogen in transfeminine individuals.^1^

At the onset of puberty, there is an increase in the amplitude and frequency of GnRH secretion in a diurnal, pulsatile manner from the hypothalamus, activating the release of the gonadotropins, luteinizing hormone (LH), and follicle-stimulating hormone (FSH) from the pituitary gland. The gonadotropins in turn trigger maturation of the gonads and the production and release of the sex steroids testosterone or estradiol (from the testes or ovaries, respectively). GnRH or GnRHa's (biosynthetic agonist of GnRH) administered in a constant rather than pulsatile manner desensitize the GnRH receptors of the anterior pituitary gland to GnRH and reversibly suppress the production of gonadotropins and subsequently, gonadal sex steroids.^2^

The two most common GnRHa's that are used within the United States for suppression of puberty in youth with gender dysphoria are leuprolide and histrelin. Leuprolide is delivered through injection ranging from monthly to every 3, 4, or 6 months.^2^ Histrelin is administered through an implant placed subcutaneously in the underside of the nondominant upper arm and releases medication for 12 months, with effectiveness to 24 months being reported.^5^ This hydrogel implant delivery system for histrelin was initially used starting in 1991 to suppress the hypothalamic–pituitary–gonadal (HPG) axis in adults with prostate cancer.

In 2007, the histrelin implant was approved for use in children with central precocious puberty (CPP).^6^

Two histrelin implants are available in the United States. Both contain 50 mg of histrelin and differ only in the amount of medication, that is, delivered daily. SupprelinLA (brand name) releases about 65 mcg/day of histrelin and has approval from the U.S. Food and Drug Administration (FDA) for treating CPP in pediatric patients. Vantas (brand name) releases about 50 mcg/day of histrelin and has FDA approval for treating advanced prostate cancer, along with other conditions that may benefit from suppressing the HPG axis in adults.

No medications carry an FDA indication for use in youth with gender dysphoria, but because of its pediatric indication for treatment of CPP, SupprelinLA is typically the brand used for this purpose. Since care for youth with gender dysphoria is often not covered by insurance plans and the cost of the SupprelinLA implant is unaffordable for most families out of pocket (costing around $45,000), medical providers have prescribed the Vantas implant (costing around $5,400) as an alternative for pubertal suppression in youth with gender dysphoria.^7^

To monitor the effectiveness of GnRHa's, clinicians rely on both physiologic response and ultrasensitive (US) assays of sex steroids and gonadotropins. Monitoring physiological changes (primarily breast development and testicular size) provide data to evaluate the clinical effectiveness of GnRHa's.^8^ However, repeated examination of these body parts can be subject to variability by provider and, more importantly, may be traumatizing for youth experiencing gender dysphoria. Checking serum hormone levels can provide a less distressing and potentially more objective approach to assess for adequacy of puberty suppression. The gold standard for biochemical monitoring of pubertal suppression has historically been a GnRH stimulation test to check responsiveness of the pituitary gland to GnRH. As this test is costly, time-consuming, and uncomfortable for patients, US LH levels are considered a useful alternative in the management of CPP.^9,10^

In the current study, by examining pre- and posttreatment random gonadotropin and sex steroid levels, we aimed to determine if SupprelinLA and Vantas were both effective in suppressing pubertal development in transgender youth being treated for gender dysphoria. To our knowledge, no research has been published examining the use or efficacy of either the SupprelinLA or Vantas implant in suppressing puberty in youth with gender dysphoria.

## Methods

Subjects were identified from two sources: existing patients with gender dysphoria receiving services at the Center for Transyouth Health and Development at Children's Hospital Los Angeles (CHLA) who were prescribed and administered a puberty-blocking implant for suppression of their endogenous puberty, and those who were enrolled as part of the Trans Youth Care study, a large observational, multisite study being conducted at CHLA/University of Southern California, Boston Children's Hospital/Harvard University, Lurie Children's Hospital of Chicago/Northwestern University, and the Benioff Children's Hospital/University of California San Francisco.

Charts of existing patients who had a histrelin implant in place were reviewed for available baseline and follow-up data. For these patients, a waiver of consent was granted. Informed consent (from parents of minors) and assent (from youth), whose data were abstracted from the larger study, were obtained in the context of the broader study.

Inclusion criteria included youth who had a histrelin implant placed at Tanner stage 2 or 3 of pubertal development for treatment of gender dysphoria. Participants were only included if they had either US gonadotropin or sex steroid levels obtained before and after histrelin implant placement. Patients who received leuprolide acetate, antiandrogens or gender-affirming hormones (estradiol or testosterone) for gender dysphoria were excluded. Those using GnRHa to treat precocious puberty as well as those who had GnRHa implants placed beyond Tanner stage 3 of pubertal development were also excluded.

Collected data were deidentified from all participants. Data were stratified and analyzed by gender identity, type of implant (Vantas or SupprelinLA), and Tanner stage at insertion of implant. US estradiol in transmasculine, and testosterone in transfeminine levels, LH, and FSH collected at baseline (T0), and then between 2 and 12 months following implant placement (T1), were abstracted from the medical record and from the larger study data pool. Hormone levels were compared across cohorts to determine if both histrelin implants had effectively induced pubertal suppression. This study received Institutional Review Board approval from all four participating sites.

Descriptive statistics were used to report demographic information, including percentages, ranges, and mean. As categorical variables (gonadotropin and sex steroid levels from T0 to T1) were found to be non-normally distributed with a Shapiro–Wilk test, Wilcoxon Signed-Rank tests were used to compare medians among paired samples (LH, FSH, estradiol, and testosterone at T0 and T1 by implant) and Mann–Whitney *U* tests were used to compare medians of independent samples' LH, FSH, estradiol, and testosterone between implants at T0 and T1. All statistical analyses utilized SPSS version 25.

## Results

### Demographics

Of the 66 eligible participants, 32 (48%) were designated male at birth and identified as transfeminine and 34 (52%) were designated female at birth and identified as transmasculine (Table 1). Twenty (30.3%) were administered the 50 mcg/day implant (Vantas), and 46 (69.7%) were administered the 65 mcg/day (SupprelinLA) implant. The majority of the participants (60.6%), identified as white, 22.7% Hispanic/Latinx, 4.5% Asian/Pacific Islander, 7.6% mixed race, and 1.5% Black/African American, with one participant declining to answer and one participant identified as “other.” The mean age at which the histrelin implant was placed was 11.3 years, with transmasculine participants starting on average younger (mean 10.8 years, range 9–15 years) compared with transfeminine participants (mean 11.8 years, range 10–15 years), consistent with the pubertal timing of the sexes.^11^

**Table 1.** Demographics

	Assigned male at birth (transfeminine) n (%)	Assigned female at birth (transmasculine) n (%)	All participants n (%)
	32 (48)	34 (52)	66 (100)
Age at implant placement mean (range)	11.8 years (10–15 years)	10.8 years (9–15 years)	11.3 years (9–15 years)
Tanner stage at implant placement	Determined by testicular volume >4 cc	Determined by breast development	
Tanner stage 2	27 (84.4)	24 (70.6)	51 (77.3)
Tanner stage 3	5 (15.6)	10 (29.4)	15 (22.7)
**Type of implant, *n* (%)**	**transfeminine**	**transmasculine**	**All participants**
SupprelinLA (65 mcg/day)	22 (68.8)	24 (70.6)	46 (69.7)
Vantas (50 mcg/day)	10 (31.3)	10 (29.4)	20 (30.3)
Ethnicity			
White	15 (46.9)	25 (73.5)	40 (60.6)
Hispanic/Latinx	12 (37.5)	3 (8.8)	15 (22.7)
Black/AA	1 (3.1)	0	1 (1.5)
API	1 (3.1)	2 (5.9)	3 (4.5)
Multiracial	1 (3.1)	4 (11.8)	5 (7.6)
Other	1 (3.1)	0	1 (1.5)
Refuse to answer	1 (3.1)	0	1 (1.5)

API, Asian/Pacific Islander.

### Changes in gonadotropin and sex steroid levels with GnRHa treatment

#### SupprelinLA

Wilcoxon Signed-Rank test indicated that median LH and FSH were significantly lower after SupprelinLA insertion at T1 (2–12 months after insertion) compared with T0 (*Z*=−4.15; *p*<0.005 and *Z*=−5.71; *p*<0.005 respectively) (Table 2). Additionally, testosterone among transfeminine and estradiol among transmasculine subjects were significantly decreased in those with a SupprelinLA implant at T1 compared with T0 (*Z*=−3.75; *p*<0.005 and *Z*=−3.58; *p*<0.005, respectively).

**Table 2.** Changes in Gonadotropin and Sex Steroid Levels After Implant Administration

	Baseline (T0)	Follow-up T1	Z	p
SupprelinLA	Median (range)	Median (range)		
LH (mIU/mL), *n*=43	0.62 (0.02–8.2)	0.2 (0.02–1.29)	−4.15	<0.001
Follicle-stimulating hormone (mIU/mL), *n*=44	2 (0.66–9.2)	0.63 (0.08–2.25)	−5.71	<0.001
Estradiol (pg/mL) (transmasculine), *n*=23	7 (2–48)	2 (1–9)	−3.75	<0.001
Total testosterone (ng/dL) (transfeminine), *n*=22	19.5 (3–365)	9 (3–28)	−3.58	<0.001
**Vantas, *n*=20**	**Baseline (T0)**	**Follow-up T1**	***Z***	***p***
LH (mIU/mL), *n*=20	1.26 (0.3–7.43)	0.21 (0.08–0.92)	−3.68	<0.001
Follicle-stimulating hormone (mIU/mL), *n*=19	3.63 (0.9–7.58)	0.5 (0.09–3.05)	−3.82	<0.001
Estradiol (pg/mL) (transmasculine), *n*=8	35.5 (18–90)	3 (2–7)	−2.52	<0.001
Total testosterone (ng/dL) (transfeminine), *n*=10	57.5 (5–334)	7 (3–13)	−2.83	<0.001

Wilcoxon signed ranks tests.

LH, luteinizing hormone.

#### Vantas

In those with a Vantas implant, Wilcoxon Signed-Rank tests indicated significant decreases in median LH and FSH at T1 compared with T0 (*Z*=−3.68; *p*<0.001 and *Z*=−3.82; *p*<0.001). Testosterone among transfeminine and estradiol among transmasculine subjects also were significantly lower at T1 compared with T0 (*Z*=−2.52; *p*<0.001 and *Z*=−2.83; *p*<0.001, respectively).

### Comparison of implants: follow-up levels of LH, FSH, and sex steroids

#### Gonadotropin levels

A Mann–Whitney test indicated that among the entire sample, there was no significant difference in median LH at T1 between those suppressed with SupprelinLA (0.2 mIU/mL) and those with Vantas (0.21 mIU/mL), *U*=386.5, *p*=0.37 (Table 3). There was also no significant difference in median FSH at T1 between those suppressed with SupprelinLA (0.63 mIU/mL) and those with Vantas (0.5 mIU/mL), *U*=419, *p*=0.9.

**Table 3.** Comparison of Implants; Baseline and Follow-up Levels of Luteinizing Hormone, Follicle-Stimulating Hormone, and Sex Steroids

All participants	SupprelinLA n=45	Vantas n=20	Z	U	p
	Median (range)	Median (range)			
LH (mIU/mL), T1	0.20 (0.02–1.29)	0.21 (0.08–0.92)	−0.9	386.5	0.37
Follicle-stimulating hormone (mIU/mL), T1	0.63 (0.08–2.25)	0.5 (0.09–3.05)	−0.125	419	0.9
**Transfeminine**	**SupprelinLA *n*=22**	**Vantas *n*=10**	*Z*	*U*	*p*
	**Median (range)**	**Median (range)**			
LH (mIU/mL), T1	0.21 (0.05–1.29)	0.36 (0.08–0.92)	−0.8	86	0.42
Follicle-stimulating hormone (mIU/mL), T1	0.37 (0.09–0.79)^a^	0.32 (0.09–0.66)	−0.7	88.5	0.49
Total testosterone (ng/dL), T1	9 (3–28)	7 (3–13)	−0.57	96.6	0.59
**Transmasculine**	**SupprelinLA *n*=24**	**Vantas *n*=10**	*Z*	*U*	*p*
	**Median (range)**	**Median (range)**			
LH (mIU/mL), T1	0.17 (0.02–0.62)	0.19 (0.08–0.64)	−0.42	109	0.7
Follicle-stimulating hormone (mIU/mL), T1	0.82 (0.08–2.25)	1.11 (0.44–3.05)^a^	−0.667	91.5	0.51
Estradiol (pg/mL), T1	2 (1–9)	4 (2–7)	−1.43	65	0.24

Mann–Whitney tests.

^a^ Two missing values.

When stratified by designated sex at birth, among transfeminine participants, there was no significant difference in median serum total testosterone observed at T1 between those youth suppressed with SupprelinLA (9 ng/dL) and those with Vantas (7 ng/dL), *U*=96.6, *p*=0.59. Among transmasculine participants, there was no significant difference in median serum estradiol at T1 between those with a SupprelinLA (2 pg/mL) and those with a Vantas (4 pg/mL), *U*=65, *p*=0.24 implant. Among all transmasculine participants, estradiol levels were within the prepubertal range at T1 (≤20 pg/mL) at T1.

### Differences in gonadotropin levels by designated sex at birth

There was no significant difference in median LH at baseline between transmasculine and transfeminine participants (0.72 mIU/mL vs. 0.62 mIU/mL, *U*=502.5, *p*=0.9) (Table 4). At T1, median LH was significantly higher in transfeminine (0.3 mIU/mL) compared with transmasculine participants (0.19 mIU/mL), *U*=365.5, *p*=< 0.001. At baseline, median FSH was not different between transfeminine (1.92 mIU/mL) and transmasculine participants (3.69 mIU/mL), *U*=237, *p*=0.34. However, at T1, transmasculine participants had significantly higher median FSH (1 mIU/mL) compared with transfeminine participants (0.34 mIU/mL), *U*=168.5, *p*<0.001. Despite these significant differences in gonadotropins at T1 between transfeminine and transmasculine participants, all participants had achieved adequate suppression into the prepubertal range at T1.

**Table 4.** Changes in Gonadotropin Levels by Gender Identity

	Transfeminine	Transmasculine	Z	U	p
LH T0 (mIU/mL), median (range)	0.72 (0.02–3.1)	0.62 (0.02–8.2)	−0.128	502.5	0.9
LH T1 (mIU/mL), median (range)	0.3 (0.05–1.29)	0.19 (0.02–0.64)	−2.12	365.5	<0.001
FSH T0 (mIU/mL), median (range)	1.92 (0.8–4.26)	3.69 (0.66–9.2)	−3.18	237	0.34
FSH T1 (mIU/mL), median (range)	0.34 (0.09–0.79)	1 (0.08–3.05)	−4.6	168.5	<0.001

FSH, follicle-stimulating hormone.

### Changes in gonadotropin and sex steroid levels by Tanner stage

Among all participants, baseline median LH was significantly higher in those participants who were suppressed in Tanner 3 (2.47 mIU/mL) versus Tanner 2 (0.55 mIU/mL), *U*=94.5, *p*<0.001 (Table 5). Similarly, baseline median FSH was significantly higher in those who were suppressed in Tanner 3 versus Tanner 2 (4.18 mIU/mL vs. 2 mIU/mL), *U*=160.5, *p*<.005. At T1, there were no longer significant differences in median LH between those who were blocked at Tanner 2 (0.2 mIU/mL) versus Tanner 3 (0.2 mIU/mL), *U*=363, *p*=0.943. While median FSH was significantly higher in those suppressed at Tanner 3 versus Tanner 2 at T1 (0.79 and 0.5 mIU/mL respectively), *U*=178, *p*=0.006, all levels were all within the prepubertal range, making this difference not clinically significant.

**Table 5.** Changes in Gonadotropin and Sex Steroid Levels by Tanner Stage and Implant Type

	Tanner 2	Tanner 3	Z	U	p
	Median (range) n=47	Median (range) n=14			
LH (mIU/mL), T0	0.55 (0.02–7.43)	2.47 (0.2–8.2)	−4.29	94.5	<0.001
LH (mIU/mL), T1	0.2 (0.02–0.94)	0.2 (0.06–1.29)	−0.071	363	0.943
FSH (mIU/mL), T0	2 (0.66–7.58)	4.18 (1.3–9.2)	−3.28	160.5	<0.001
FSH (mIU/mL), T1	0.5 (0.08–1.8)	0.79 (0.23–3.05)	−2.79	178	0.006
**Transfeminine**	**Tanner 2**	**Tanner 3**	***Z***	***U***	***p***
	**Median (range) *n*=27**	**Median (range) *n*=5**			
Testosterone (ng/dL), T0	19 (3–166.1)	166 (28–365)	−2.62	17	0.006
Testosterone (ng/dL), T1	7 (3–23)	11 (7–28)	−2.22	25	0.026
**Transmasculine**	**Tanner 2**	**Tanner 3**	***Z***	***U***	***p***
	Median (range)	Median (range)			
Estradiol (pg/mL), T0	8.5 (2–69) *n*=23	35.5 (6–90) *n*=9	−3.04	31	0.002
Estradiol (pg/mL), T1	2 (1–9) *n*=22	4 (2–7) *n*=8	−2.743	37.5	0.016

Mann–Whitney tests.

Among transfeminine participants, median total testosterone was higher at baseline in those who were suppressed at Tanner 3 (166 ng/dL) versus Tanner 2 (19 ng/dL), *U*=17, *p*=0.006. At T1, median total testosterone remained higher in Tanner 3 participants (11 ng/dL) versus Tanner 2 (7 ng/dL), *U*=25, *p*=0.026, but all T1 levels were in the prepubertal to early pubertal range. Among transmasculine participants, median estradiol was higher at baseline in those suppressed at Tanner 3 (35.5 ng/dL) versus Tanner 2 (8.5 ng/dL), *U*=31, *p*=0.002. At T1, while median estradiol in those suppressed in Tanner 3 (4 ng/dL) was higher than those suppressed in Tanner 2 (2) *U*=37.5, *p*=0.016, all participants achieved prepubertal to early pubertal levels.

## Discussion

Development of unwanted, permanent secondary sex characteristics in youth can be traumatic, trigger or intensify gender dysphoria along with other mental health conditions, and may necessitate future surgical intervention for transgender individuals. The introduction of puberty suppression to pause, or avoid altogether the development of irreversible secondary sex characteristics is perhaps the most significant intervention in transgender care in the last 30 years. Unfortunately, central puberty suppression with GnRHa's is largely unaffordable for families without insurance coverage, or those with insurance plans that do not cover interventions for gender dysphoria.^7^

The histrelin implant indicated for pediatric use in patients with CPP (SupprelinLA) is markedly more expensive than the implant indicated for use in adults with prostate cancer (Vantas). Additionally, the use of either of these GnRHa's for gender dysphoria youth is off label, which poses an additional barrier to accessing this critical intervention.^7^

This article demonstrates that both Vantas and SupprelinLA are equally effective at reducing gonadotropin and hormone levels into a pre- or early pubertal range. The difference in gonadotropin levels between transmasculine and transfeminine participants reflect known patterns; FSH predominance in those individuals designated female at birth and LH predominance in those designated male at birth.^12^

Overall, both implants were successful at central suppression of the HPG axis. Gonadotropins and sex steroid levels achieved by SupprelinLA in this sample of youth with gender dysphoria at T1 were similar to those reported for youth administered SupprelinLA for treatment of CPP.^9^ Gonadotropin and testosterone levels in this sample of youth with gender dysphoria treated with Vantas were comparable to studies monitoring nontransgender men administered Vantas for symptomatic treatment of prostate cancer.^13,14^

Limitations of this study include a relatively small sample size. Future studies with larger sample sizes will assist in validating these findings. Additionally, these data reflect a variable follow-up period given the retrospective study design; future prospective studies addressing the long-term effectiveness and safety of these implants will be useful. Laboratory evaluation differed among participants, resulting in variable parameters for prepubertal levels.

## Conclusions

In this study, treatment with a histrelin implant (Vantas or SupprelinLA) was effective in suppressing gonadotropins and sex steroids in early-to-mid-pubertal youth with gender dysphoria. These data may inform decisions about insurance coverage of Supprelin and/or Vantas for youth with gender dysphoria. Future studies examining long-term effectiveness, safety, and side effects of these implants will further inform these decisions.

## Abbreviations Used

API: Asian/Pacific Islander
CHLA: Children's Hospital Los Angeles
CPP: central precocious puberty
FDA: Food and Drug Administration
FSH: follicle-stimulating hormone
GnRHa: gonadotropin-releasing hormone agonists
HPG: hypothalamic–pituitary–gonadal
LH: luteinizing hormone
US: ultrasensitive
